# Supplementary material for: Quercetin Alleviates Endoplasmic Reticulum Stress-Induced Apoptosis in Buffalo Ovarian Granulosa Cells
Source: Animals (Basel). 2022 Mar 20;12(6):787. doi: 10.3390/ani12060787 (PMC8944572; doi:10.3390/ani12060787)
Supplement: Supplementary file 1 [file animals-12-00787-s001.zip › animals-1598941-supplementary.pdf]

Supplementary Information

Table S1. The primers sequences for q-PCR. *PERK* (Genebank: 102406027), *IRE-1* (102404660), *EIF-2A* (Genebank:102389500), *ATF4* (Genebank:102393344), *ATF6*(Genebank:102394003), *BCL-2*(Genebank:102402077), *BAX*(Genebank:102400076), *Caspase3* (Genebank:102415768), *Caspase9* (Genebank:102412012).

Table S1. Primer sequence.

Primer	Primer sequence
PERK-F	ATCAGCACTTTAGATGGCCG
PERK-R	GGTTTGCTAAGGCTGGATGA
IRE1-F	TTACGATCCCTGAGTTGGTG
IRE1-R	ATAACGTACCAAATGTCCTGC
EIF-2A-F	GGAACAGACGCTGCACTAC
EIF-2A-R	GCTTTGGCAGGCATGAACC
ATF-4-F	AAACCCTACGACCCTCCTG
ATF-4-R	GCCCTCTTCTTCTGGCGGTA
ATF-6-F	GAACCTCAGCCACTTTCGC
ATF-6-R	CTGAGGAGGGGTTATTGGAGC
BCL-2-F	GACCCGACGGCAGGAGAA
BCL-2-R	CCCAAGATGGCTGGAATGAG
BAX-F	GCAAACTGGTGCTCAAGGC
BAX-R	GAGCACTCCAGCCACAAGA
Caspase3-F	AATACCAGTTGAGGCAGA
Caspase3-R	GTCCGAGAACACTCTACG
Caspase9-F	CCTACGTCCTGAATGCAGACCC
Caspase9-R	GTGCTCAGCCCCGAGTCCC

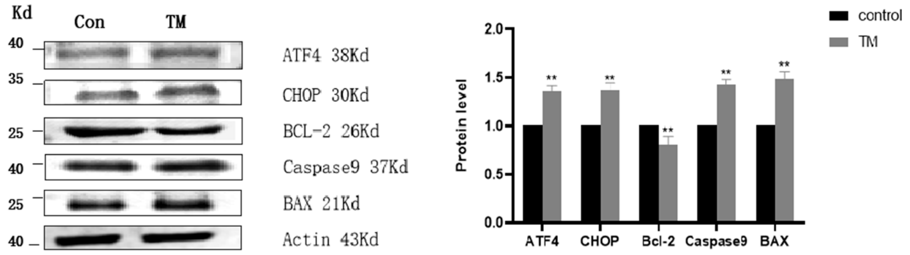

Figure S1. Figure 2C Western blot densitometric readings. Effect of TM on ER stress and apoptosis-related proteins. (**A**) (**left**) Representative western blot of TM treatment of GCs after 48 h. (**right**) Densitometry analysis of the western blot in A. The asterisks refer to the level of significance (* $p < 0.05$, ** $p < 0.01$).

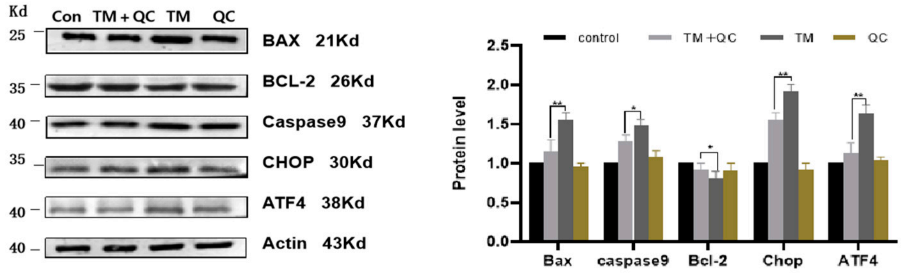

Figure S2. Figure 4B Western blot densitometric readingsEffect of quercetin pre-treatment on ER stress and apoptosis-related proteins. (A) (**left**) Representative western blot of QC pre-treatment of GCs after 8 hours. (**right**) Densitometry analysis of the western blot in A. The asterisks refer to the level of significance (* $p < 0.05$, ** $p < 0.01$).
